# Supplementary material for: Investigating the role of undercoordinated Pt sites at the surface of layered PtTe2 for methanol decomposition
Source: Nat Commun. 2024 Jan 22;15:653. doi: 10.1038/s41467-024-44840-z (PMC10803346; doi:10.1038/s41467-024-44840-z)
Supplement: Supplementary file 1 — Supplementary Information [file 41467_2024_44840_MOESM1_ESM.pdf]

Supplementary Information

Investigating the Role of Undercoordinated Pt Sites at the Surface of Layered $PtTe_2$ for Methanol Decomposition

Jing-Wen Hsueh[1], Lai-Hsiang Kuo[1], Po-Han Chen[2], Wan-Hsin Chen[3], Chi-Yao Chuang[3], Chia-Nung Kuo[4,5], Chin-Shan Lue[4,5,6], Yu-Ling Lai[7], Bo-Hong Liu[7], Chia-Hsin Wang[7], Yao-Jane Hsu[7], Chun-Liang Lin,[3,*] Jyh-Pin Chou[8,*] and Meng-Fan Luo[1,*]

[1]Department of Physics, National Central University, No. 300 Jhongda Rd., Jhongli District, Taoyuan City, 320317, Taiwan

[2]Department of Materials Science and Engineering, National Tsing Hua University, 101, Section 2 Kuang-Fu Road, Hsinchu 300044, Taiwan

[3]Department of Electrophysics, National Yang Ming Chiao Tung University, No. 1001 University Rd., Hsinchu 300039, Taiwan

[4]Department of Physics, National Cheng Kung University, No. 1 University Rd., Tainan 701, Taiwan

[5]Taiwan Consortium of Emergent Crystalline Materials, Ministry of Science and Technology, Taipei 10601, Taiwan

[6]Program on Key Materials, Academy of Innovative Semiconductor and Sustainable Manufacturing, National Cheng Kung University, Tainan 701, Taiwan

[7]National Synchrotron Radiation Research Center, No.101 Hsin-Ann Rd., Hsinchu Science Park, Hsinchu 300092, Taiwan

[8]Department of Physics, National Changhua University of Education, No. 1, Jin-De Rd., Changhua 50007, Taiwan

Corresponding authors:

mfl28@phy.ncu.edu.tw (Meng-Fan Luo); jpchou@cc.ncue.edu.tw (Jyh-Pin Chou); clin@nycu.edu.tw (Chun-Liang Lin)

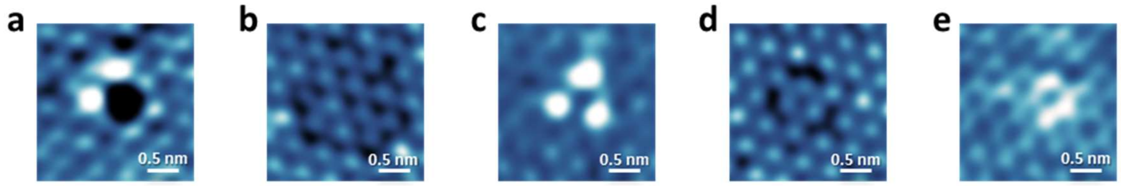

Figure	Type of defects	% of total defects
Fig. S1(a)	Te vacancy in the top row	37.8%
Fig. S1(b)	Pt vacancy in the second layer	21.6%
Fig. S1(c)	Te vacancy in the bottom row	20.9%
Fig. S1(d)	Pt vacancy in the first layer	13.5%
Fig. S1(e)	Antisites defect with Te replacing Pt	6.1%

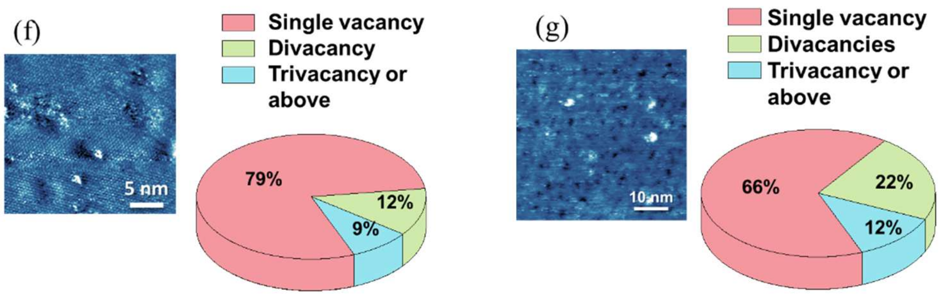

Figure S1. (a)-(e) Five types of surface defects at as-cleaved $PtTe_2$ surface. STM images were acquired at 77 K in constant-current mode (V_s = -50.7 mV, I_t = 2.7 nA) using an electrochemically etched tungsten tip. These surface defects, as described in the table, were also observed in previous STM studies.[1] Comparison of the densities and sizes of Te vacancies produced by small (f) and larger (g) Ar^+ dosages. It is clear that the number of Te vacancies and the fraction of larger vacancies increase with Ar^+ dosages.

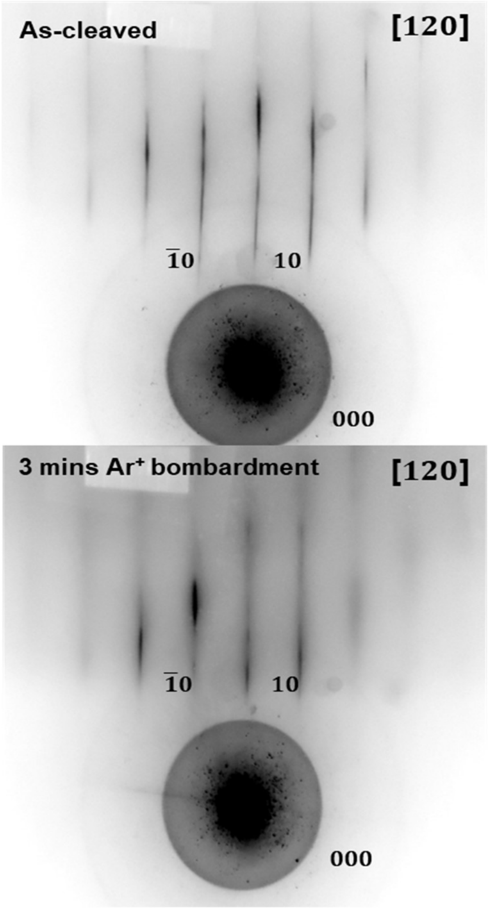

Figure S2. Comparison of RHEED patterns obtained from $PtTe_2$ before and after Ar^+ bombardment (3 mins). The RHEED measurements were performed with an incident electron beam of 25 keV at a grazing angle of 2° to 3° relative to the surface, and the patterns were obtained at the [120] azimuth of $PtTe_2$ substrate. The sharp and bright reflection rods from as-cleaved $PtTe_2$ (top panel) suggest a highly crystalline $PtTe_2$ surface; the patterns correspond to a hexagonal lattice with a lattice constant of 3.97 Å, quite close to the value obtained from STM (Figure 1). The reflection rods became slightly blurred and faint (bottom pattern) after Ar^+ bombardment, suggesting attenuated surface crystallinity — the extent of ordered $PtTe_2$ structures decreased. Nevertheless, the still clear diffraction patterns indicate that the surface crystallinity remains to a great extent, although the corresponding STM image (Figure 3c) cannot resolve the atomic arrangement. The RHEED patterns from the samples with smaller Ar^+ dosages remain also clear.

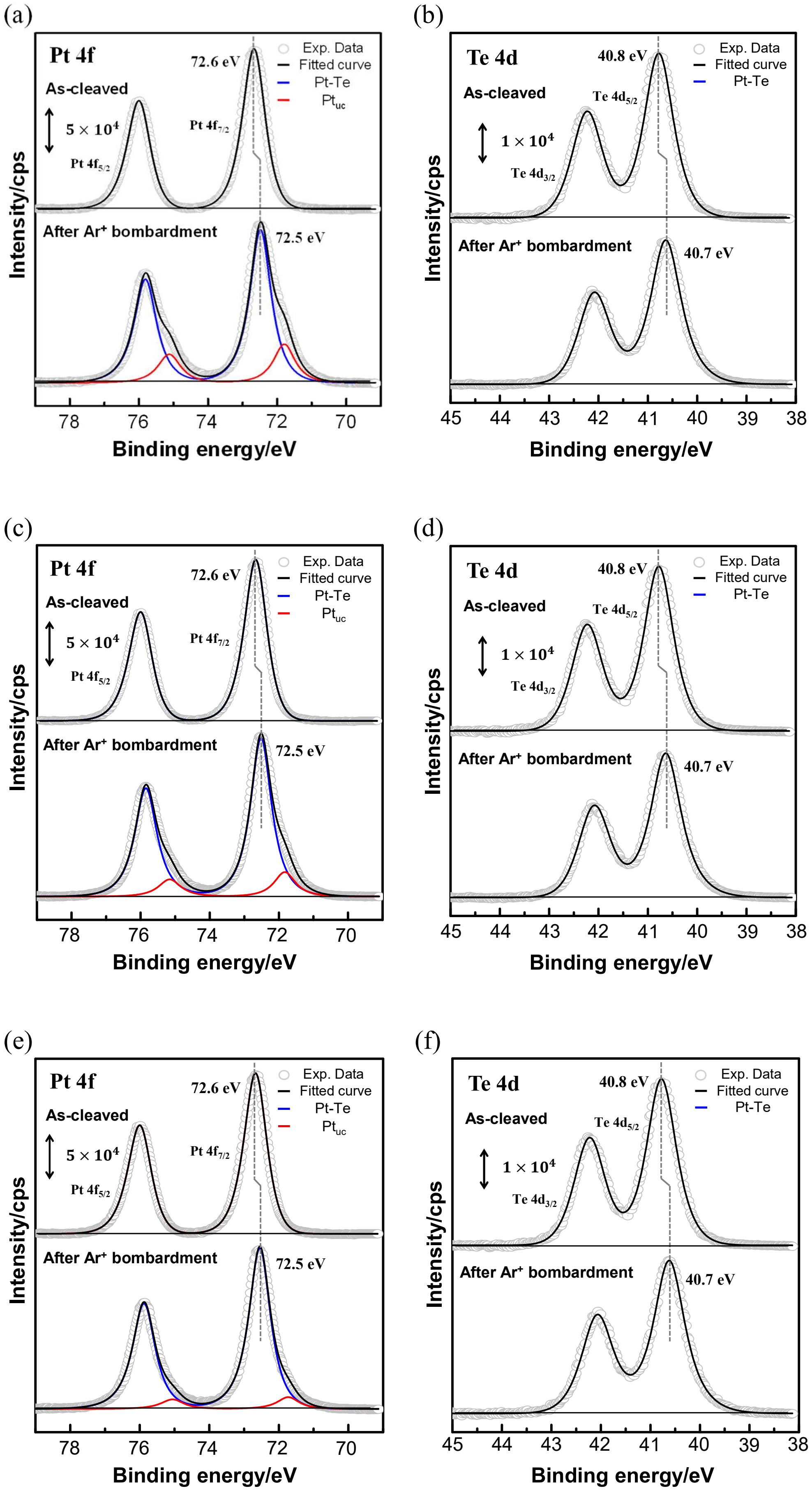
(a)
Pt 4f
As-cleaved
72.6 eV
5 × 10^4
Pt 4f7/2
Pt 4f5/2
After Ar+ bombardment
72.5 eV
Exp. Data
Fitted curve
Pt-Te
Ptuc
Intensity/cps
Binding energy/eV
78 76 74 72 70
(b)
Te 4d
As-cleaved
40.8 eV
Te 4d5/2
Te 4d3/2
1 × 10^4
After Ar+ bombardment
40.7 eV
Exp. Data
Fitted curve
Pt-Te
Intensity/cps
Binding energy/eV
45 44 43 42 41 40 39 38
(c)
Pt 4f
As-cleaved
72.6 eV
5 × 10^4
Pt 4f7/2
Pt 4f5/2
After Ar+ bombardment
72.5 eV
Exp. Data
Fitted curve
Pt-Te
Ptuc
Intensity/cps
Binding energy/eV
78 76 74 72 70
(d)
Te 4d
As-cleaved
40.8 eV
Te 4d5/2
Te 4d3/2
1 × 10^4
After Ar+ bombardment
40.7 eV
Exp. Data
Fitted curve
Pt-Te
Intensity/cps
Binding energy/eV
45 44 43 42 41 40 39 38
(e)
Pt 4f
As-cleaved
72.6 eV
5 × 10^4
Pt 4f7/2
Pt 4f5/2
After Ar+ bombardment
72.5 eV
Exp. Data
Fitted curve
Pt-Te
Ptuc
Intensity/cps
Binding energy/eV
78 76 74 72 70
(f)
Te 4d
As-cleaved
40.8 eV
Te 4d5/2
Te 4d3/2
1 × 10^4
After Ar+ bombardment
40.7 eV
Exp. Data
Fitted curve
Pt-Te
Intensity/cps
Binding energy/eV
45 44 43 42 41 40 39 38

Figure S3. PES spectra of Pt 4f and Te 4d core levels from layered $PtTe_2$ as bombarded by Ar^+ (0.5 keV) for (a,b) 9, (c,d) 4 and (e,f) 2 mins. Gray circles denote the spectra and black lines the sum of fitted curves; the signals from intact Pt and under-coordinated Pt (Pt_{uc}) in the layered $PtTe_2$ are fitted with blue and red lines, respectively. The spectral features for varied Ar^+ dosages are similar despite varied intensities of the Pt_{uc} signals.

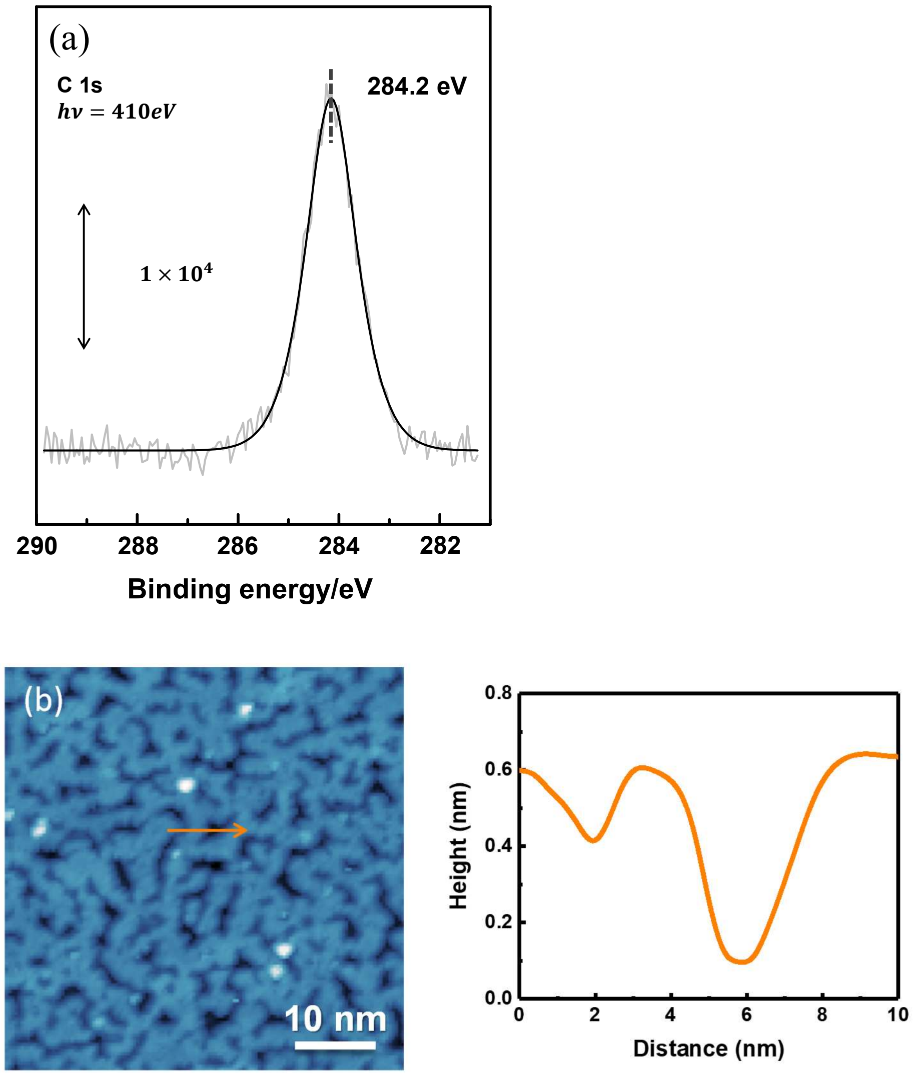

Figure S4. (a) A PES spectrum of C 1s core level from layered $PtTe_2$ annealed to 750 K for 1 hour. (b) STM image (V_s = -150 mV, I_t = 1.20 nA) for layered $PtTe_2$ bombarded with a Ar^+ dosage near 4 in Figure 2c. In (a), the gray and black lines denote the experimental data and fitted curve, respectively. The distinct feature centered at 284.2 eV is assigned to atomic carbons on $PtTe_2$ surface. The surface atomic carbons appear typically after annealing above 500 K. The inset in (b) shows a lateral profile across two vacancies. The depth near 0.5 nm corresponds to the thickness of a $PtTe_2$ bilayer. The result reflects the early stage of the formation of $PtTe_2$ islands (patches) with an edge height about 0.5 nm. The white spots in the image indicate the Pt-Te nanoclusters formed by nucleation of redeposited Pt and Te.

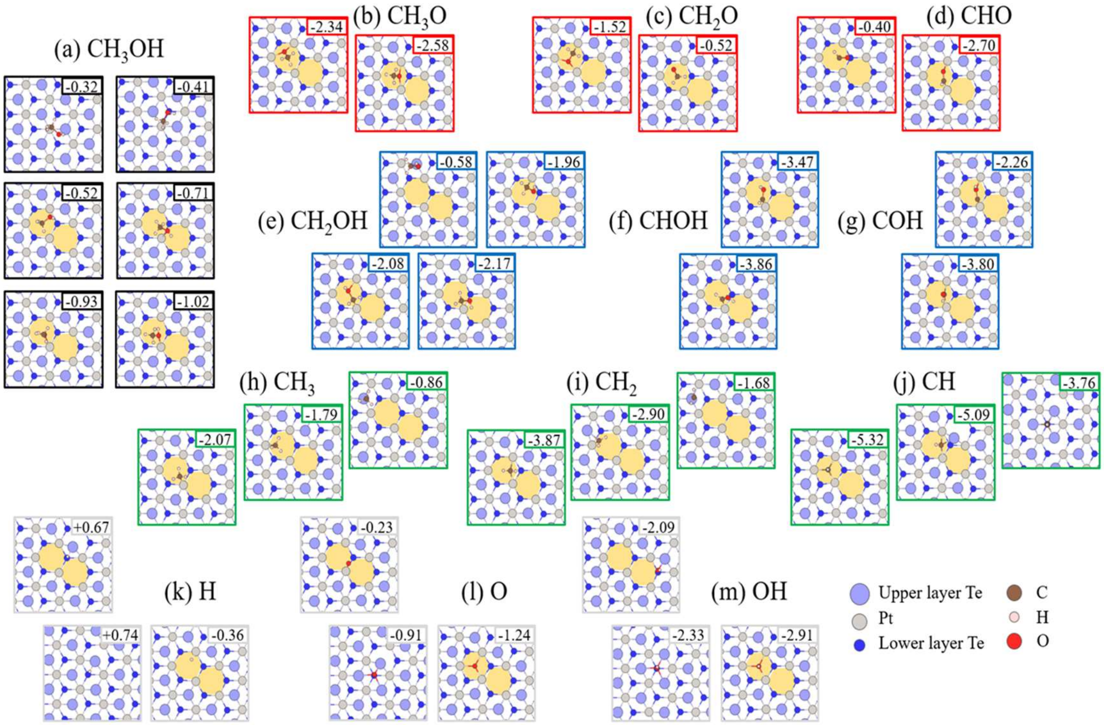

Figure S5. Varied adsorption configurations of methanol and its decomposition intermediates or fragments in the Te divacancy model. For the sake of clarity, the Te-divacancy site is shadowed with orange. The value at the top-right corner of each graph is provided for the corresponding adsorption energy.

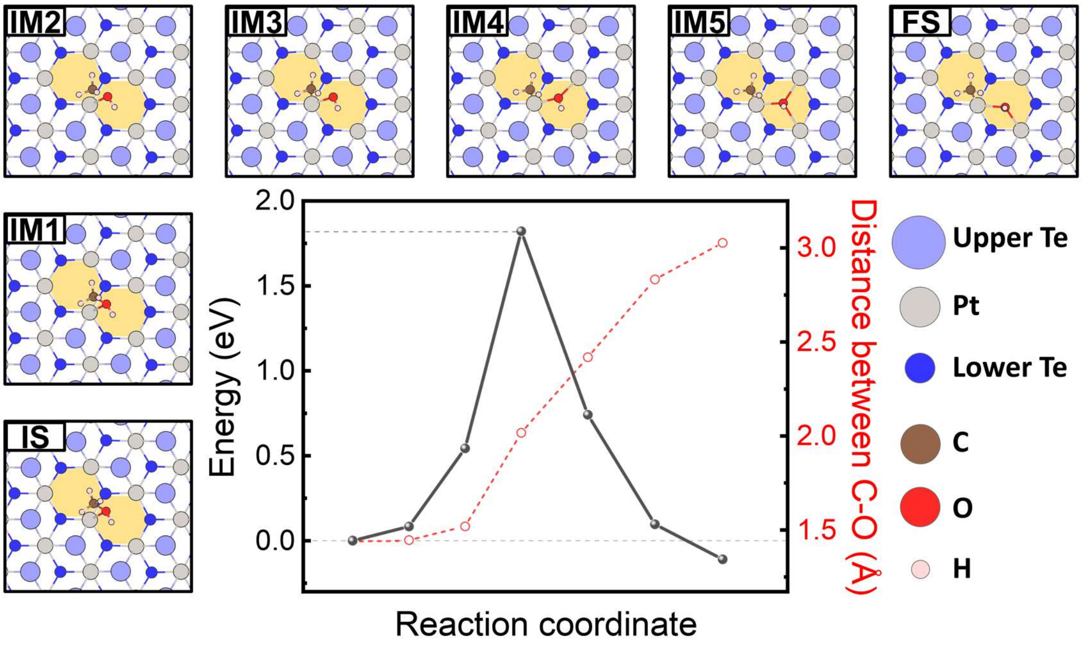

Figure S6. The energy profile of methanol (CH_3OH) C-O bond scission on the Te-divacancy site (shadowed with orange). The energy barrier is 1.82 eV. Details of initial state (IS), CINEB images (IM) and final state (FS) are presented. The red dashed line indicates the variation of the C-O distance.

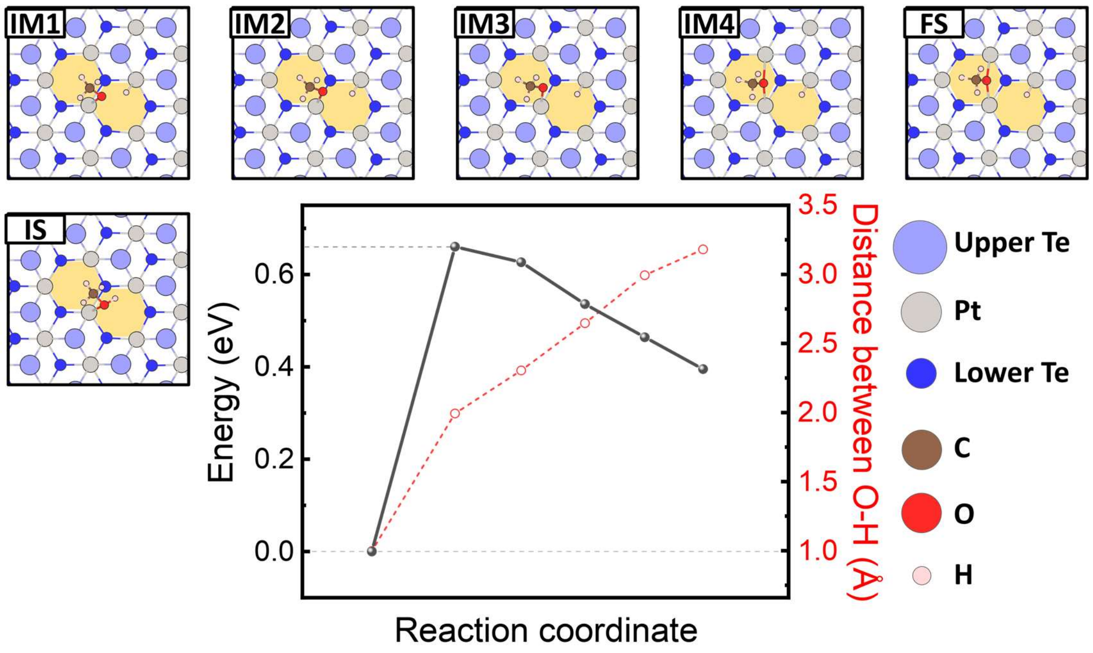

Figure S7. The energy profile of dehydrogenation of methanol (CH_3OH) to methoxy (CH_3O) on the Te-divacancy site (shadowed with orange). The energy barrier is 0.66 eV. Details of initial state (IS), CINEB images (IM) and final state (FS) are presented. The red dashed line indicates the variation of the O-H distance.

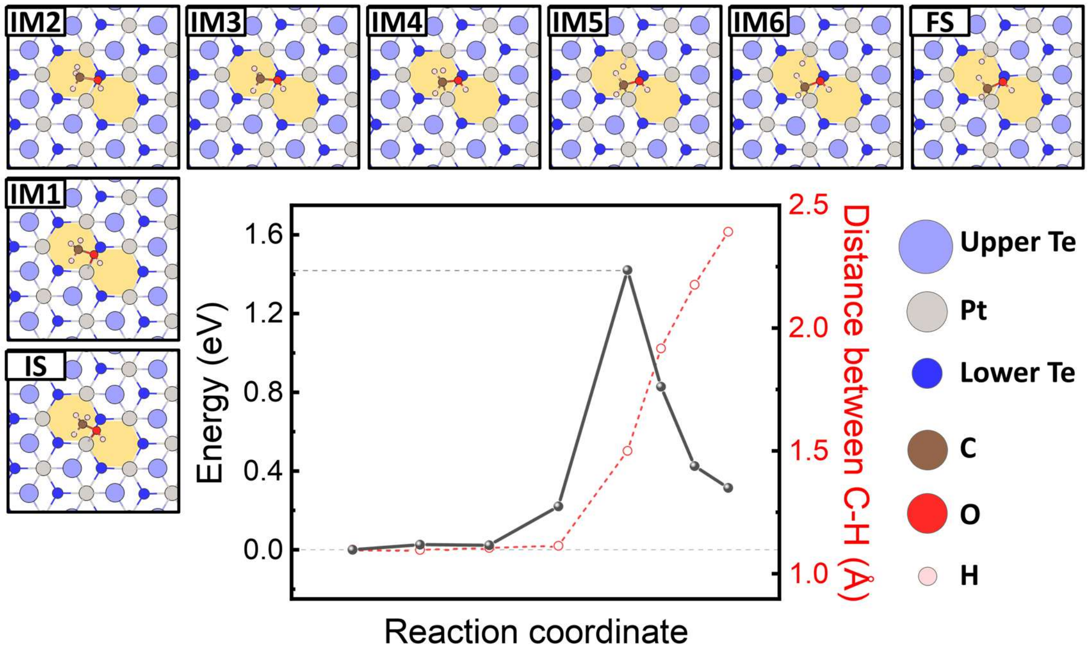

Figure S8. The energy profile of dehydrogenation of methanol (CH_3OH) to hydroxymethyl (CH_2OH) on the Te-divacancy site (shadowed with orange). The energy barrier is 1.42 eV. Details of initial state (IS), CINEB images (IM) and final state (FS) are presented. The red dashed line indicates the variation of the C-H distance.

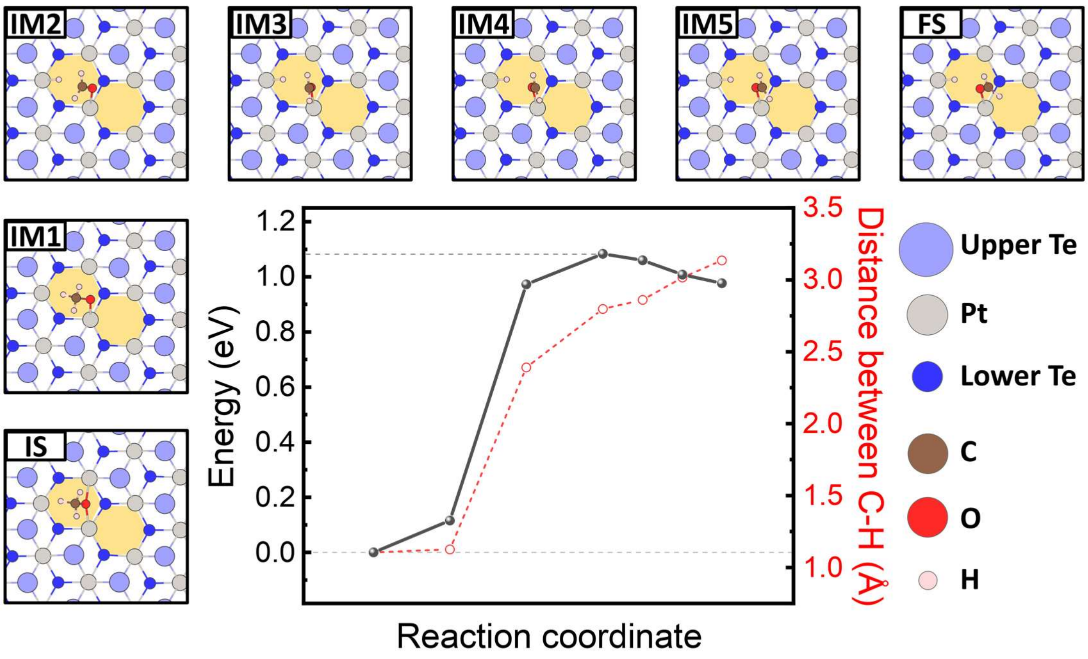

Figure S9. The energy profile of dehydrogenation of methoxy (CH_3O) to formaldehyde (CH_2O) on the Te-divacancy site (shadowed with orange). The energy barrier is 1.08 eV. Details of initial state (IS), CINEB images (IM) and final state (FS) are presented. The red dashed line indicates the variation of the C-H distance.

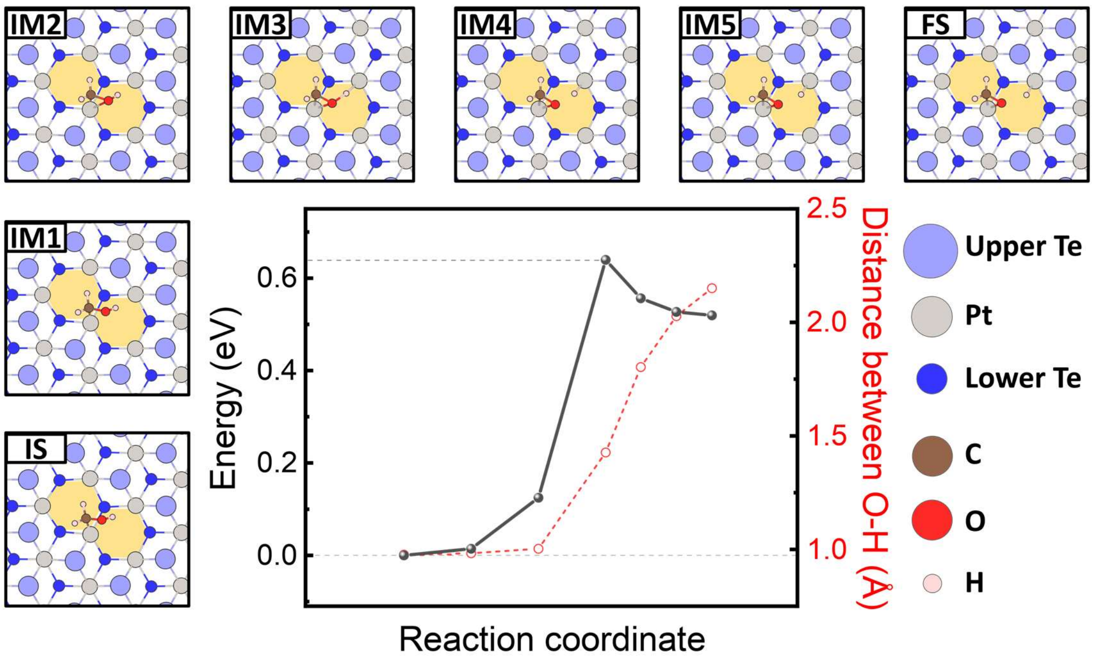

Figure S10. The energy profile of dehydrogenation of hydroxymethyl (CH_2OH) to formaldehyde (CH_2O) on the Te-divacancy site (shadowed with orange). The energy barrier is 0.64 eV. Details of initial state (IS), CINEB images (IM) and final state (FS) are presented. The red dashed line indicates the variation of the O-H distance.

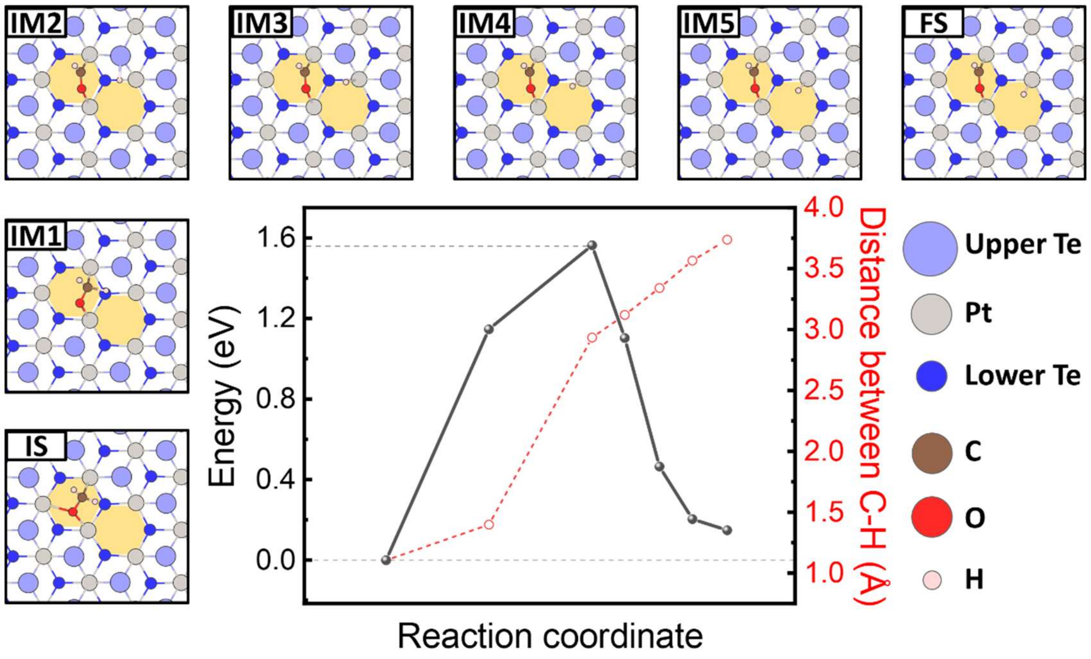

Figure S11. The energy profile of dehydrogenation of formaldehyde (CH_2O) to CHO on the Te-divacancy site (shadowed with orange). The energy barrier is 1.56 eV. Details of initial state (IS), CINEB images (IM) and final state (FS) are presented. The red dashed line indicates the variation of the C-H distance.

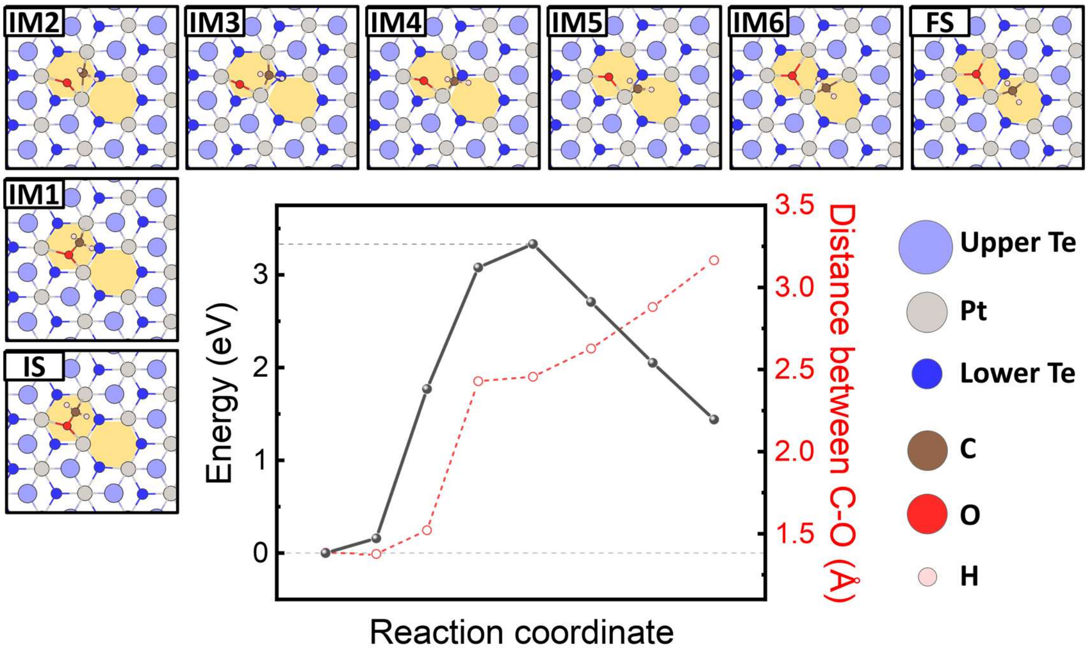

Figure S12. The energy profile of formaldehyde (CH_2O) C-O bond scission on the Te-divacancy site (shadowed with orange). The energy barrier is 3.33 eV. Details of initial state (IS), CINEB images (IM) and final state (FS) are presented. The red dashed line indicates the variation of the C-O distance.

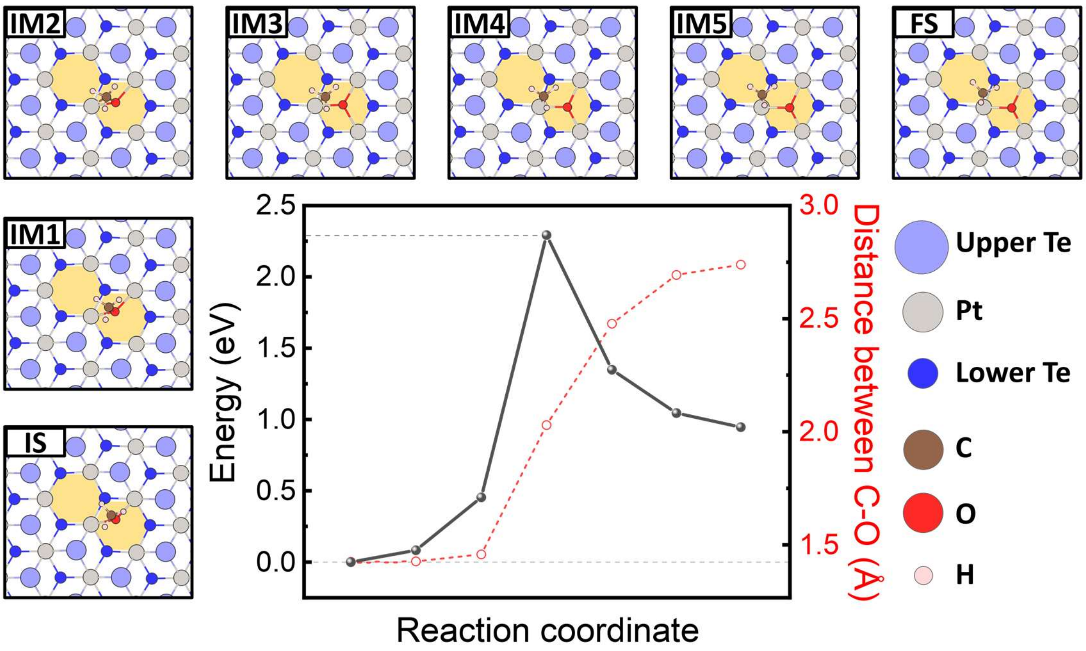

Figure S13. The energy profile of methoxy (CH_3O) C-O bond scission on the Te-divacancy site (shadowed with orange). The energy barrier is 2.29 eV. Details of initial state (IS), CINEB images (IM) and final state (FS) are presented. The red dashed line indicates the variation of the C-O distance.

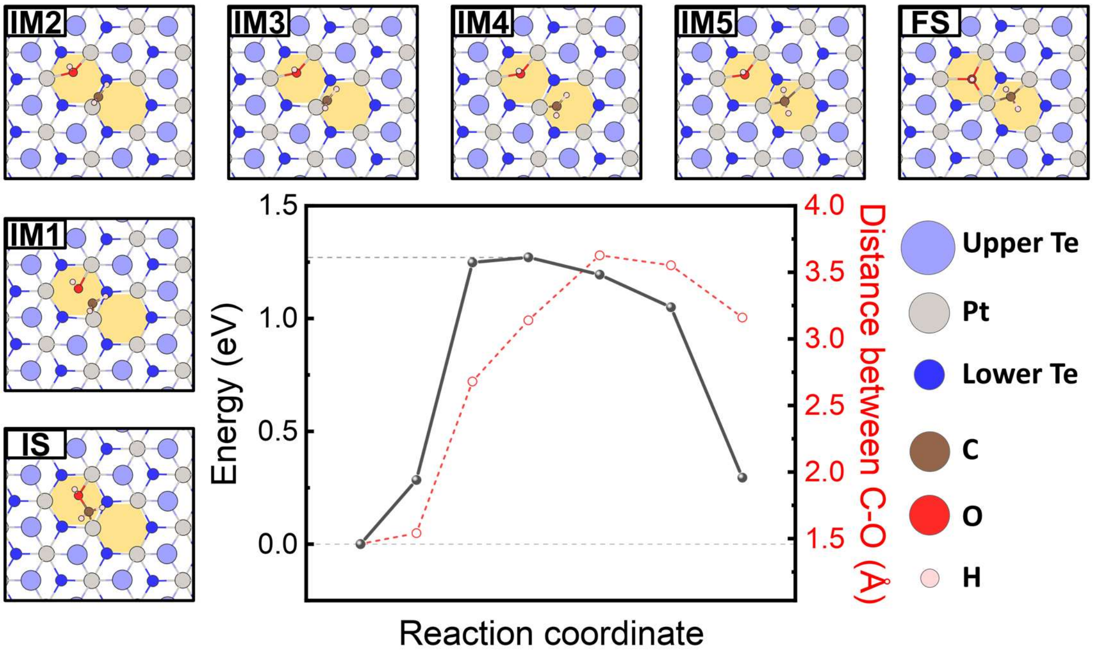

Figure S14. The energy profile of hydroxymethyl (CH_2OH) C-O bond scission on the Te-divacancy site (shadowed with orange). The energy barrier is 1.27 eV. Details of initial state (IS), CINEB images (IM) and final state (FS) are presented. The red dashed line indicates the variation of the C-O distance.

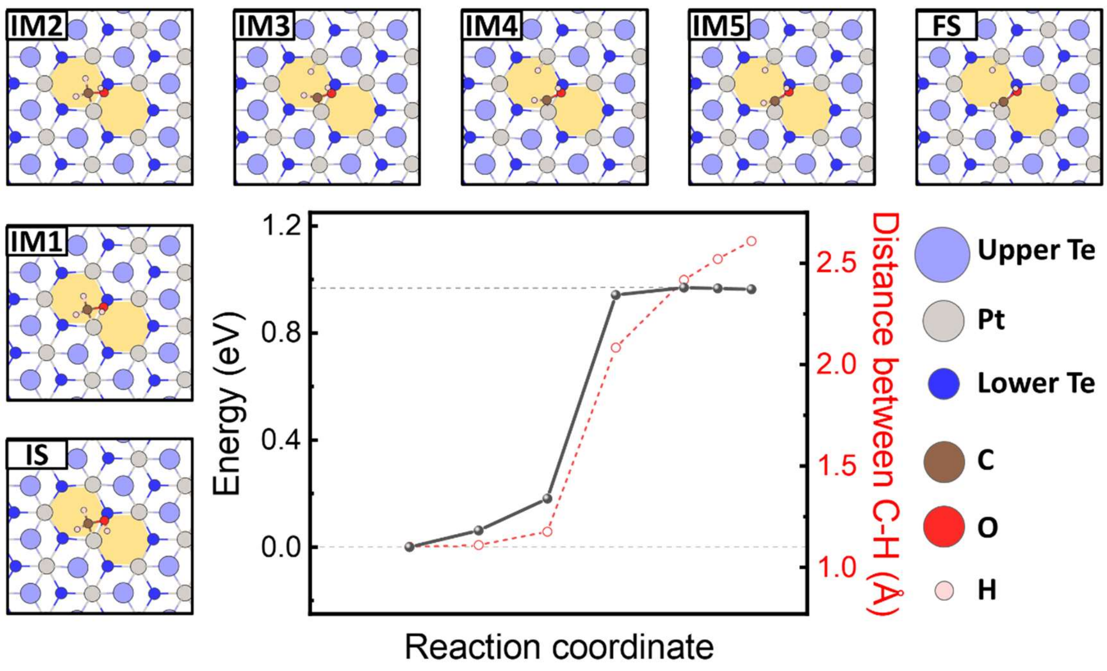

Figure S15. The energy profile of dehydrogenation of hydroxymethyl (CH_2OH) to CHOH on the Te-divacancy site (shadowed with orange). The energy barrier is 0.97 eV but the inverse process ($CHOH^* + H^* \rightarrow CH_2OH^*$) has a negligible barrier and CH_2OH^* has a lower total energy. Details of initial state (IS), CINEB images (IM) and final state (FS) are presented. The red dashed line indicates the variation of the C-H distance.

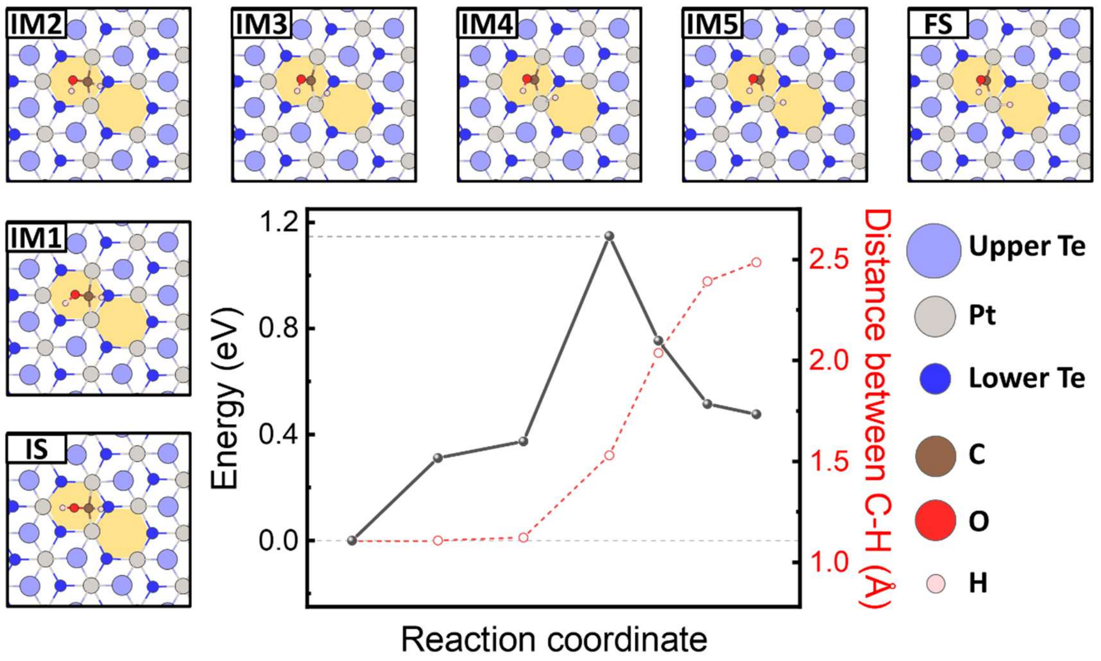

Figure S16. The energy profile of dehydrogenation of CHOH to COH on the Te-divacancy site (shadowed with orange). The energy barrier is 1.15 eV. Details of initial state (IS), CINEB images (IM) and final state (FS) are presented. The red dashed line indicates the variation of the C-H distance.

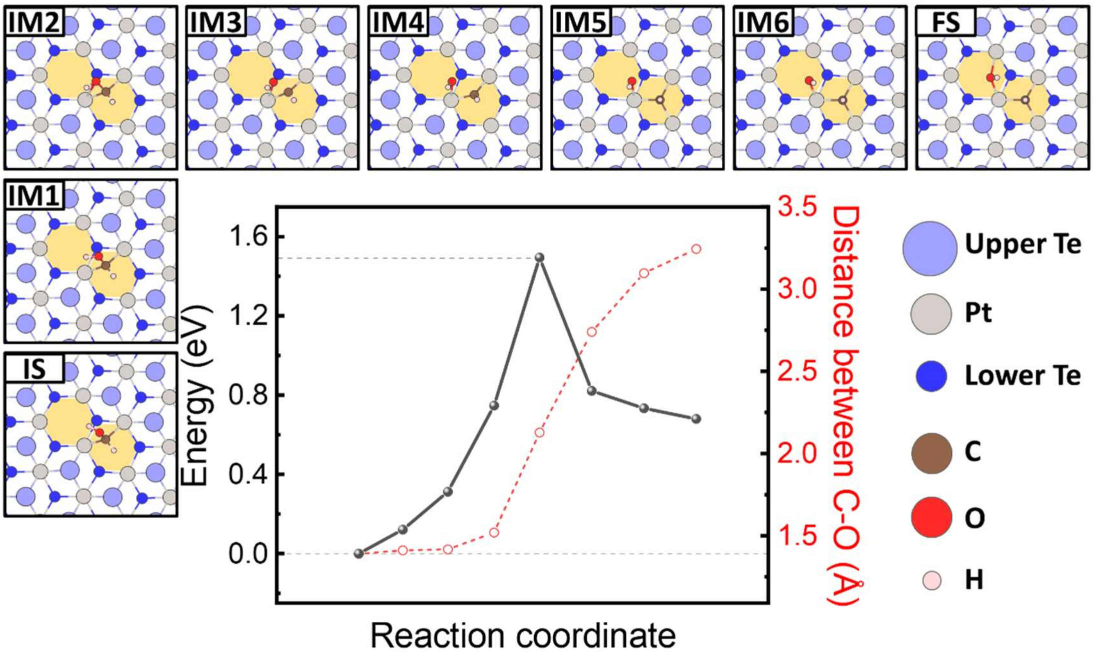

Figure S17. The energy profile of CHOH C-O bond scission on the Te-divacancy site (shadowed with orange). The energy barrier is 1.49 eV. Details of initial state (IS), CINEB images (IM) and final state (FS) are presented. The red dashed line indicates the variation of the C-O distance.

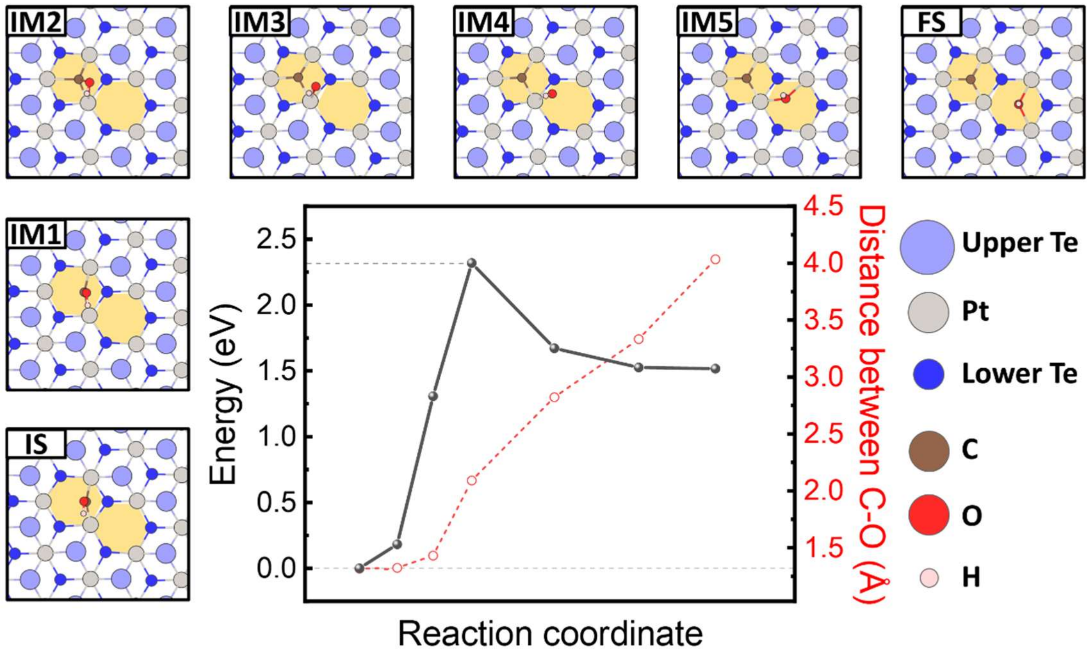

Figure S18. The energy profile of COH C-O bond scission on the Te-divacancy site (shadowed with orange). The energy barrier is 2.32 eV. Details of initial state (IS), CINEB images (IM) and final state (FS) are presented. The red dashed line indicates the variation of the C-O distance.

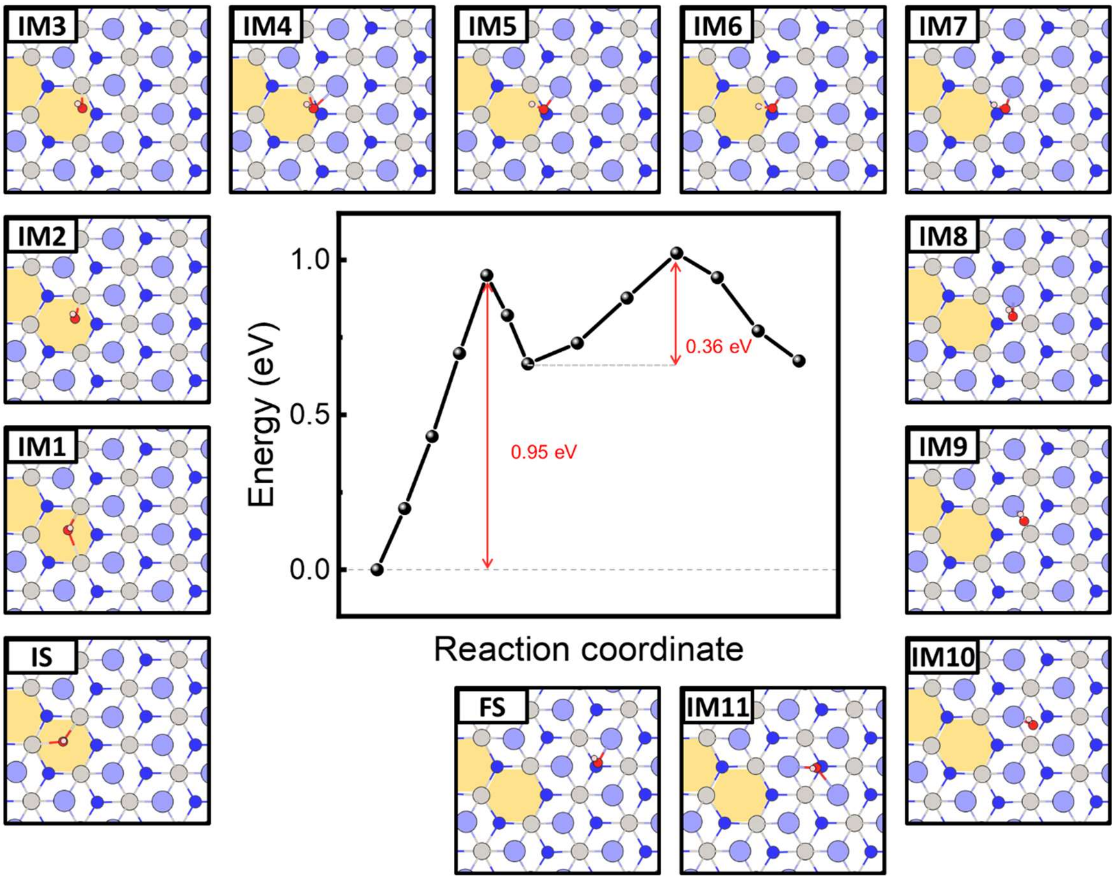

Figure S19. The energy profile of hydroxyl (OH) diffusion from the divacancy defect site to the $PtTe_2$ basal plane. The energy barriers are indicated (red) in the figure. Details of initial state (IS), CINEB images (IM), and final state (FS) are presented.

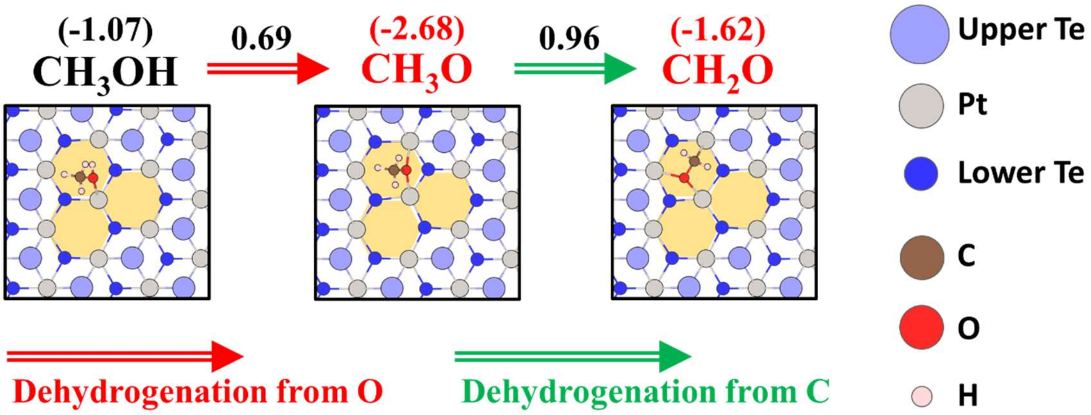

Figure S20. Dehydrogenation of methanol (CH_3OH) to formaldehyde (CH_2O) in the Te-trivacancy model. The adsorbed methanol first dehydrogenates to methoxy (CH_3O) and then to formaldehyde (CH_2O). The processes have energy barriers of 0.69 and 0.96 eV, respectively. The detailed information on the energy barrier calculations is provided in Figures S20 and S21. Also, the adsorption energies of related species are calculated and indicated in the parentheses, with the adsorption configurations shown in the panels.

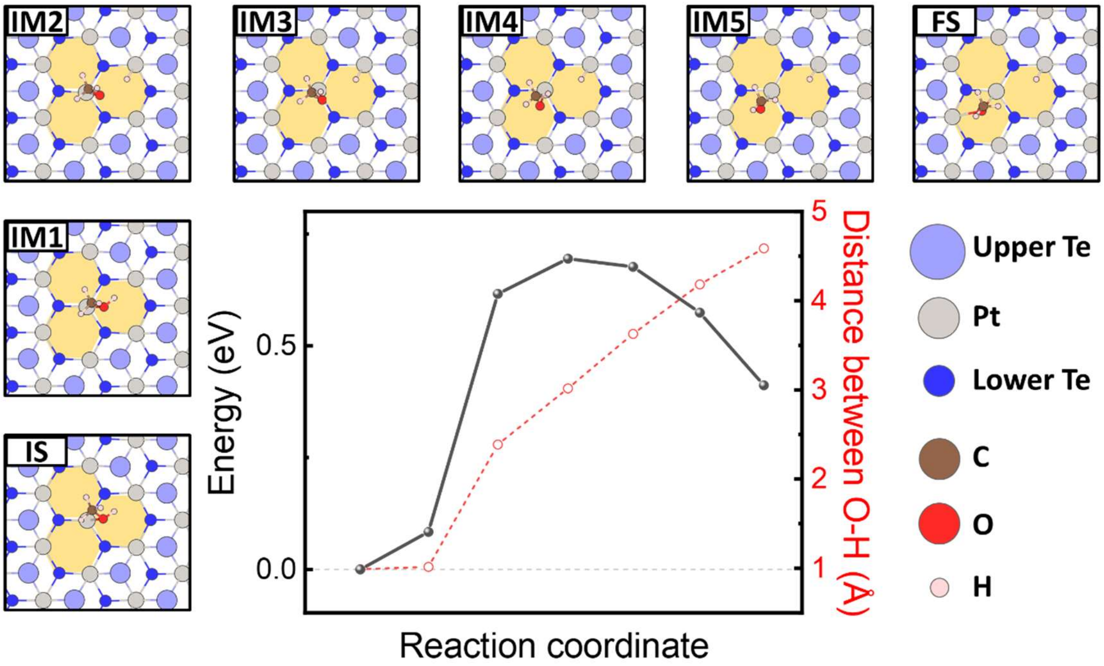

Figure S21. The energy profile of dehydrogenation of methanol (CH_3OH) to methoxy (CH_3O) on the Te-trivacancy site (shadowed with orange). The energy barrier is 0.69 eV. Details of initial state (IS), CINEB images (IM) and final state (FS) are presented. The red dashed line indicates the variation of the O-H distance.

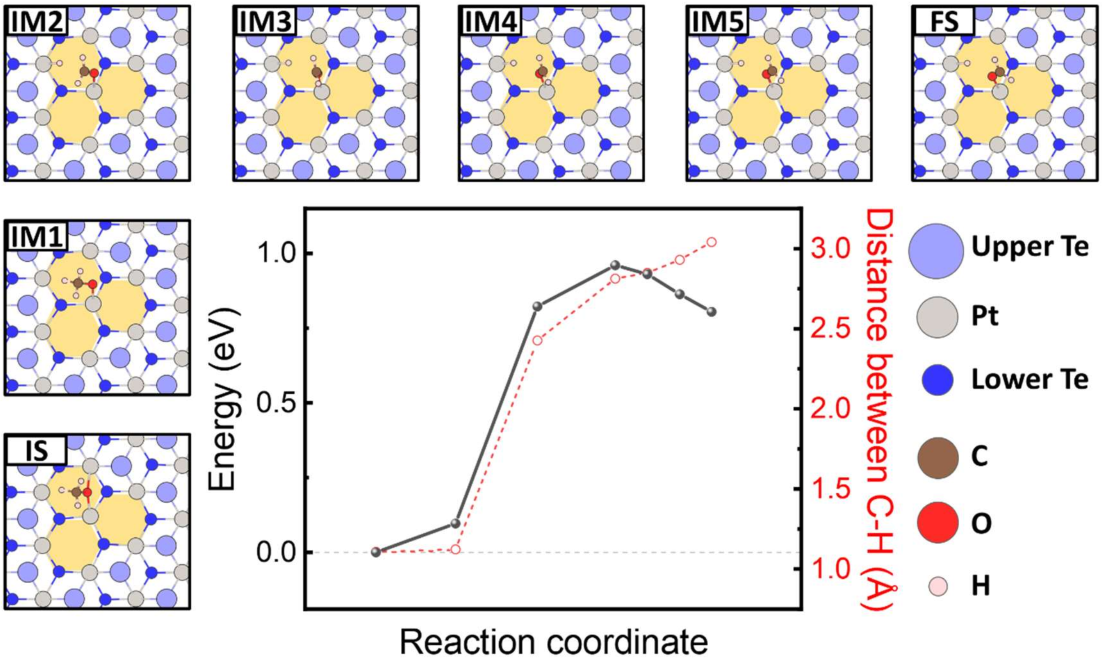

Figure S22. The energy profile of dehydrogenation of methoxy (CH_3O) to formaldehyde (CH_2O) on the Te-trivacancy site (shadowed with orange). The energy barrier is 0.96 eV. Details of initial state (IS), CINEB images (IM) and final state (FS) are presented. The red dashed line indicates the variation of the C-H distance.

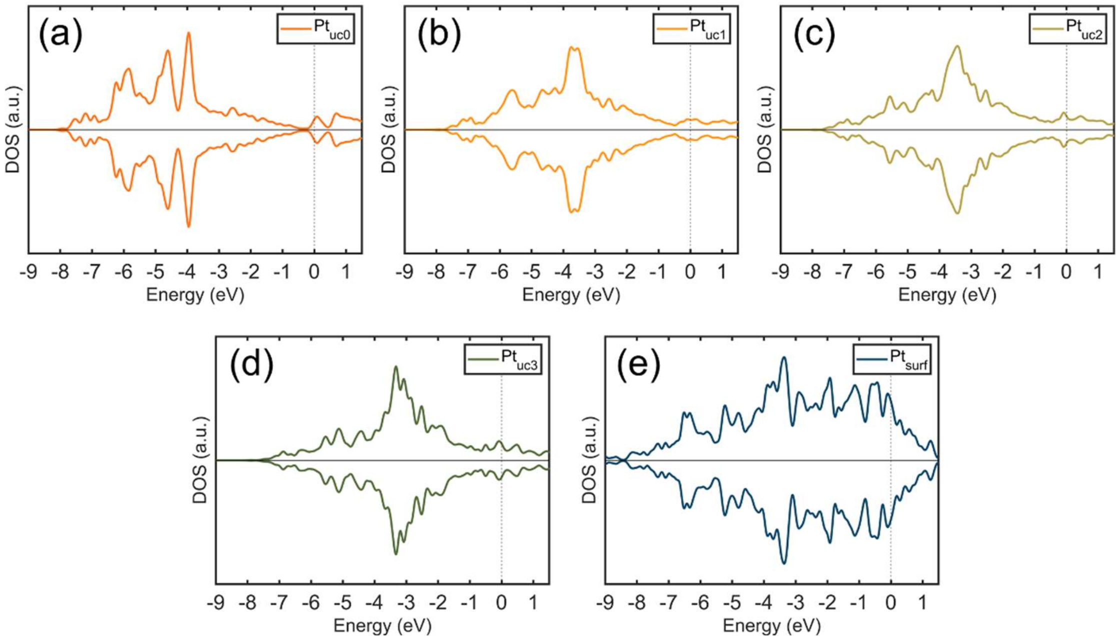

Figure S23. Comparison of local density of states (LDOS) of (a)-(d) Pt_{uc0-3} at $PtTe_2$ surface and (e) Pt at Pt(111) surface. Pt_{uc0} stands for the Pt in structurally perfect $PtTe_2$, Pt_{uc1-3} stands for Pt with 1-3 missing Te-Pt bonds (coordination number 5-3) at $PtTe_2$ surface. The result in (e) was obtained from a Pt(111) surface model — a (1×1) supercell with 7 Pt layers.

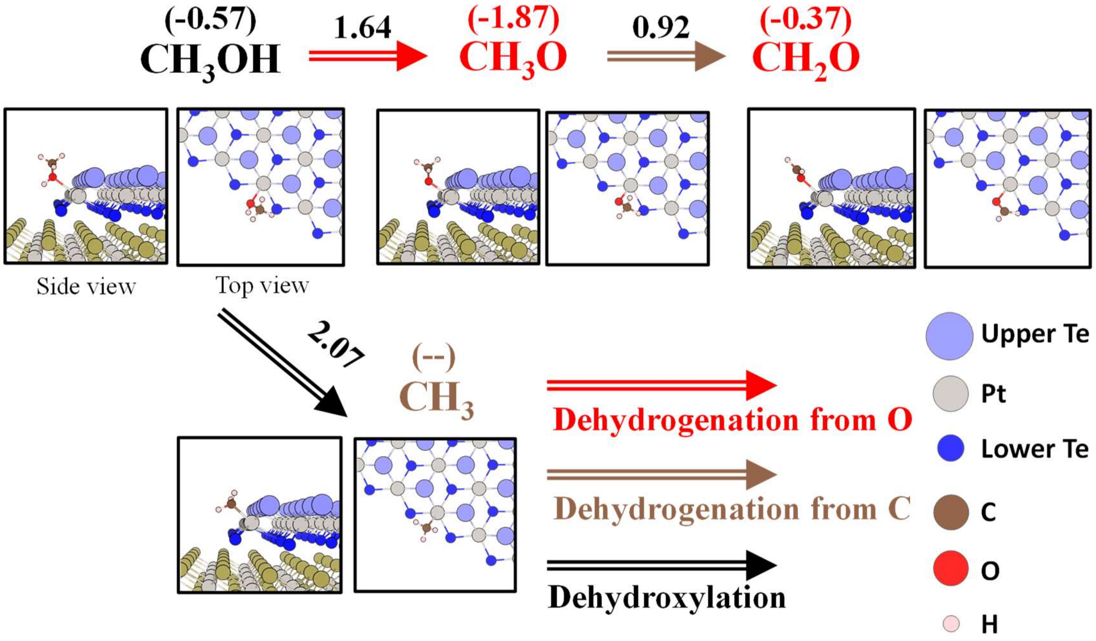

Figure S24. Dehydrogenation and dihydroxylation of methanol (CH_3OH) on the edge sites of a $PtTe_2$ island. The adsorbed methanol first dehydrogenates to methoxy (CH_3O) and then to formaldehyde (CH_2O); the processes have energy barriers of 1.64 and 0.92 eV, respectively. Alternatively, the methanol decomposes via C-O bond scission to yield CH_3 and OH, with an energy barrier of 2.07 eV. The detailed information on the energy barrier calculations is provided in Figures S25 - S27. Also, the adsorption energies of related species are calculated and indicated in the parentheses, with the adsorption configurations shown in the panels. In the light of a much smaller barrier for desorption (0.57 eV), the methanol would prefer desorption to decomposition. Details about this edge-site model can be found in Ref. 2.

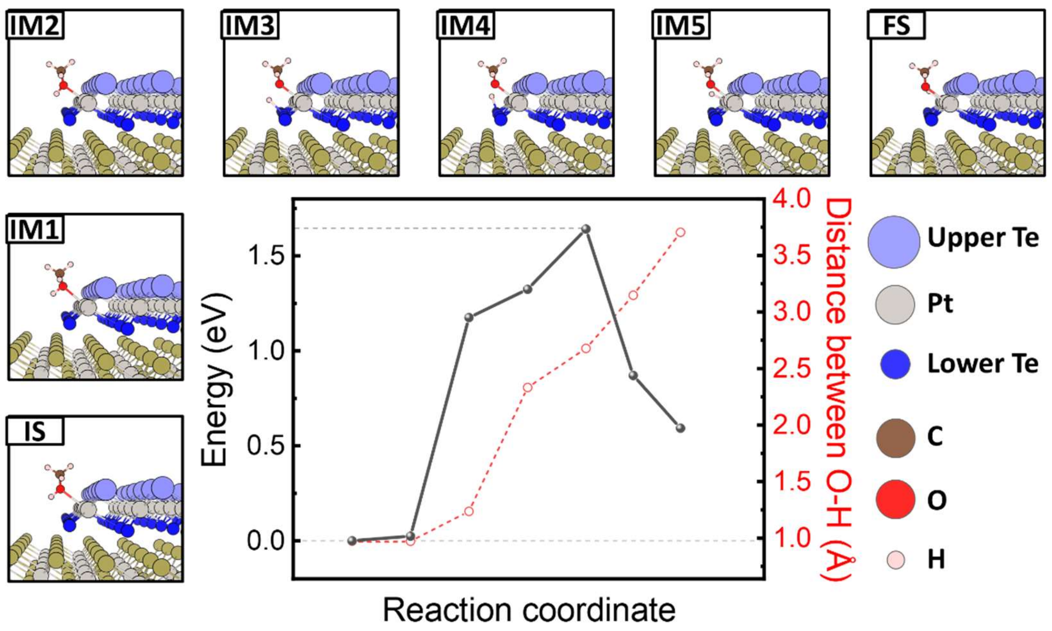

Figure S25. The energy profile of dehydrogenation of methanol (CH_3OH) to methoxy (CH_3O) on the edge sites of a $PtTe_2$ island. The energy barrier is 1.64 eV. Details of initial state (IS), CINEB images (IM) and final state (FS) are presented. The red dashed line indicates the variation of the O-H distance.

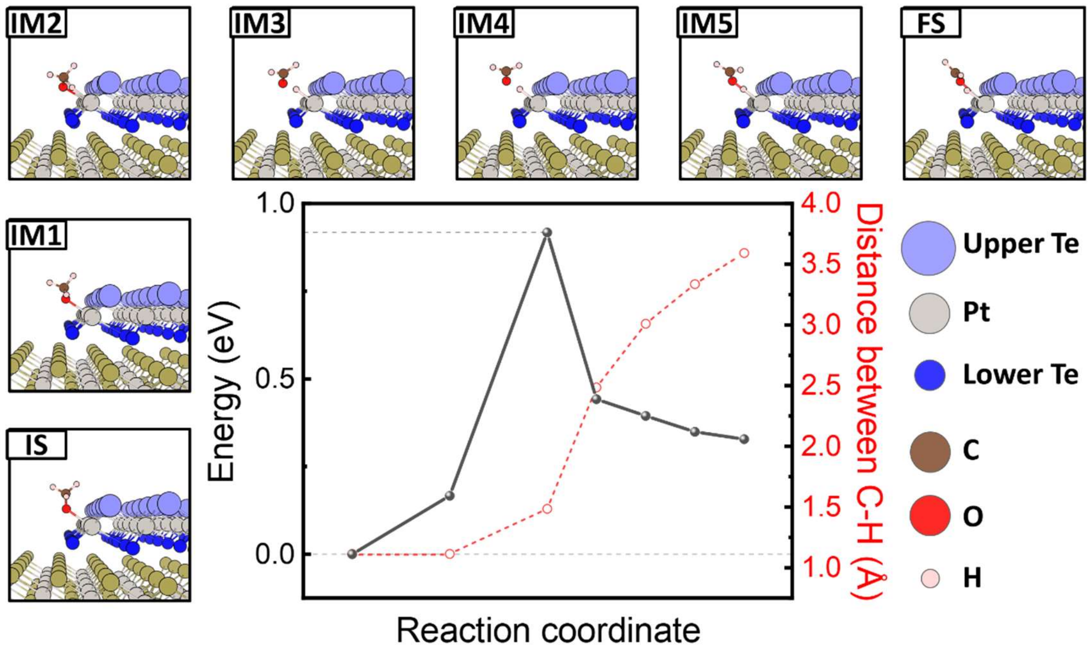

Figure S26. The energy profile of dehydrogenation of methoxy (CH_3O) to formaldehyde (CH_2O) on the edge sites of a $PtTe_2$ island. The energy barrier is 0.92 eV. Details of initial state (IS), CINEB images (IM) and final state (FS) are presented. The red dashed line indicates the variation of the C-H distance.

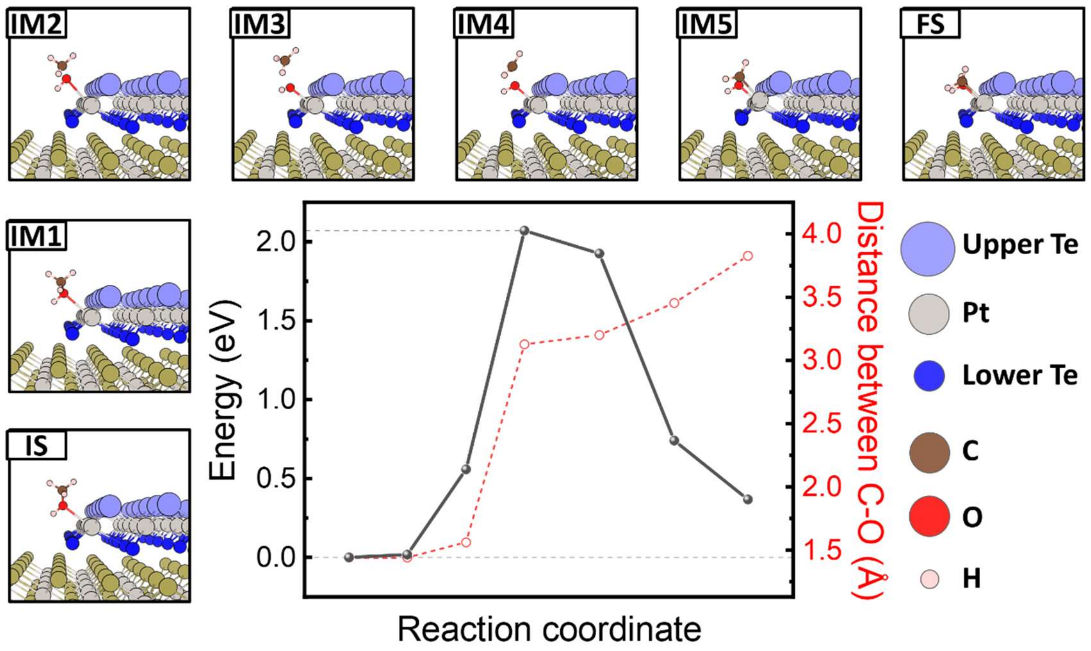

Figure S27. The energy profile of CH_3OH C-O bond scission on the edge sites of a $PtTe_2$ island. The energy barrier is 2.07 eV. Details of initial state (IS), CINEB images (IM) and final state (FS) are presented. The red dashed line indicates the variation of the C-O distance.

Supplementary References

1. Zhussupbekov K*, et al.* Imaging and identification of point defects in $PtTe_2$. *npj 2D Materials and Applications* **5**, 14 (2021).

2. Li J, Joseph T, Ghorbani-Asl M, Kolekar S, Krasheninnikov AV, Batzill M, Edge and Point-Defect Induced Electronic and Magnetic Properties in Monolayer $PtSe_2$. *Advanced Functional Materials* **32**, 2110428 (2022).
